# Explainable machine learning integrating CT-derived intratumoral heterogeneity for histological subtype prediction in pure ground-glass lung adenocarcinoma: multicenter validation and transcriptomic characterization

**DOI:** 10.3389/fmolb.2026.1958127

**Published:** 2026-10-12

**Authors:** Ying Deng, Jiajun Liu, Dinghua Li, Bingyi Zhang, Bin Xiang, Wanyin Qi, Sanhong Zhang

**Affiliations:** 1 Department of Ultrasound, Wuhan Fourth Hospital, Wuhan, China; 2 Hengyang Medical School, University of South China, Hengyang, Hunan, China; 3 Department of Radiology, The Central Hospital of Xiangtan (The Affiliated Hospital of Hunan University), Xiangtan, Hunan, China; 4 Department of Radiology, The Affiliated Hospital of Southwest Medical University, Luzhou, Sichuan, China; 5 Liuyang Traditional Chinese Medicine Hospital, Changsha, Hunan, China

**Keywords:** computed tomography, explainable machine learning, intratumoral heterogeneity, lung adenocarcinoma, pure ground-glass nodule, radiogenomics, transcriptomics

## Abstract

**Background:**

Preoperative differentiation of lepidic-predominant adenocarcinoma from other histological subtypes in lung adenocarcinoma presenting as pure ground-glass nodules remains challenging. We evaluated whether a CT-derived intratumoral heterogeneity score provides complementary predictive and biological information beyond routine clinical and CT features.

**Methods:**

This retrospective multicenter study included 533 patients, divided into a training cohort (n = 373) and a held-out validation cohort (n = 160). Twelve supervised algorithms were compared exclusively within the training cohort using stratified five-fold cross-validation and grid-search optimization; predictors, hyperparameters, and the decision threshold were locked before the held-out cohort was evaluated once. Incremental value of the ITH score and leave-one-center-out internal–external validation were additionally assessed. Segmentation reproducibility was evaluated in 100 lesions. An independent radiogenomic cohort of 43 pathological Tis/T1 lung adenocarcinomas was analyzed by differential-expression and Hallmark gene-set enrichment analyses after median ITH-score stratification.

**Results:**

XGBoost achieved the highest training-set cross-validation AUC (0.908 ± 0.021) and was selected before held-out evaluation. In the validation cohort, the AUC was 0.919 (95% CI, 0.870–0.968), with accuracy 0.850, precision 0.857, sensitivity 0.545, specificity 0.966, and Brier score 0.098. Adding the ITH score to clinical and CT features increased AUC from 0.867 to 0.919 (ΔAUC = 0.052; DeLong P = 0.012), increased PR-AUC from 0.731 to 0.824, and reduced Brier score from 0.126 to 0.098. Leave-one-center-out AUCs ranged from 0.824 to 0.882. The ITH score showed good-to-excellent repeat-segmentation reproducibility. In the radiogenomic cohort, a high ITH score was associated with coordinated enrichment of G2M checkpoint (NES = 1.86, FDR <0.001), E2F targets (NES = 1.86, FDR <0.001), and epithelial–mesenchymal transition (NES = 1.55, FDR = 0.001).

**Conclusion:**

CT-derived ITH provided incremental information for histological subtype prediction and was associated with proliferation-, cell-cycle-, and EMT-related transcriptional programs. The moderate sensitivity at the prespecified operating point and the retrospective design warrant prospective external validation before clinical implementation.

## Introduction

The increasing detection of pure ground-glass nodules (pGGNs) has created an important need to distinguish relatively indolent from biologically higher-risk lung adenocarcinomas before surgery. pGGNs may represent adenocarcinoma *in situ*, minimally invasive adenocarcinoma, or invasive adenocarcinoma with different predominant growth patterns (Fu et al., 2021; Lee et al., 2019; Zuo et al., 2026c). Among invasive tumors, lepidic-predominant adenocarcinoma (LPA) generally has more favorable outcomes than acinar-, papillary-, micropapillary-, or solid-predominant disease (Kadota et al., 2014; Song et al., 2013; Hung et al., 2014). Imaging-based predictions may therefore provide useful preoperative context, but they should complement rather than replace definitive pathological assessment.

Routine CT characteristics, including tumor size, mean CT attenuation, shape, vacuole sign, and pleural indentation, provide useful clues to invasiveness and histological composition (Lee et al., 2018; Zhang et al., 2021; Aherne et al., 2018). Radiomics extends visual assessment by quantifying intensity, texture, and shape across the whole tumor, and multicenter studies have demonstrated its value for histological grading and invasive phenotypes in lung adenocarcinoma (Fan et al., 2026; Qi et al., 2026). However, whole-tumor summaries can average spatially distinct phenotypes. Habitat-based approaches address this limitation by partitioning tumors into radiologically distinct subregions and quantifying within-tumor variation, but their interpretation remains centered on subregional phenotypes rather than a unified description of spatial organization across the entire lesion (Zuo et al., 2025b). The ITH score was developed to bridge these scales by combining local radiomic variation with the global organization of connected imaging phenotypes. A higher score reflects greater fragmentation of connected imaging regions and should be interpreted as an imaging phenotype rather than a direct measurement of genomic clones or cellular diversity (Li et al., 2023).

Previous studies have associated ITH-derived imaging phenotypes with invasiveness, high-grade patterns, and histological subtype in lung adenocarcinoma, while more recent multicenter studies have extended the framework to lymphovascular invasion and prognosis (Zhang et al., 2024; Zheng et al., 2024; Zuo et al., 2025a; Zuo et al., 2025b; Zhao Y. et al., 2026; Zuo et al., 2026a; Zuo et al., 2026e). Nevertheless, three questions remain important. First, model selection must be separated rigorously from held-out evaluation to avoid optimistic bias. Second, the incremental value and segmentation reproducibility of the ITH score require direct evaluation. Third, the biological interpretation of an imaging-derived heterogeneity score remains incomplete without independent molecular characterization.

We therefore developed an explainable machine-learning model for preoperative histological subtype prediction and used SHAP to interpret the contribution and direction of the included predictors. We then performed an independent transcriptomic characterization to examine whether higher- and lower-ITH-score phenotypes were associated with coordinated molecular programs.

## Materials and methods

### Study participants and ethics approval

This retrospective multicenter study included patients from three institutions: The Affiliated Hospital of Southwest Medical University, Luzhou, Sichuan, China; Liuyang Traditional Chinese Medicine Hospital, Changsha, Hunan, China; and The Central Hospital of Xiangtan (The Affiliated Hospital of Hunan University), Xiangtan, Hunan, China. The study was conducted in accordance with the Declaration of Helsinki and was approved by the respective institutional ethics committees. Approval was obtained from the Medical Ethics Committee of Liuyang Traditional Chinese Medicine Hospital (Opinion No. 2022-016), the Medical Ethics Committee of Xiangtan Central Hospital (Approval No. 2023-08-18), and the Medical Ethics Committee of Southwest Medical University (Approval No. SWMU-IRB-2024-068). The Southwest Medical University ethics documentation explicitly included an application for waiver of informed consent for the retrospective analysis. All patient data used for analysis were anonymized before analysis.

The study cohort comprised 533 patients with stage IA lung adenocarcinoma presenting as pGGNs. Patients were eligible if they had a pathologically confirmed diagnosis and complete preoperative thin-section chest CT images with a reconstructed section thickness of 0.625–1.25 mm. Exclusion criteria were multifocal lung adenocarcinoma, rare histological subtypes such as colloid, fetal, or invasive mucinous adenocarcinoma, receipt of chemotherapy or radiotherapy before CT examination or surgery, and inadequate CT image quality. After eligibility assessment, the pooled multicenter cohort was stratified according to histological outcome and randomly divided in a 7:3 ratio into a training cohort (n = 373) and a held-out internal validation cohort (n = 160). The held-out cohort was not used for model selection or hyperparameter optimization and was evaluated only after the final model had been locked. The overall analytical workflow is shown in Figure 1.

**FIGURE 1:**
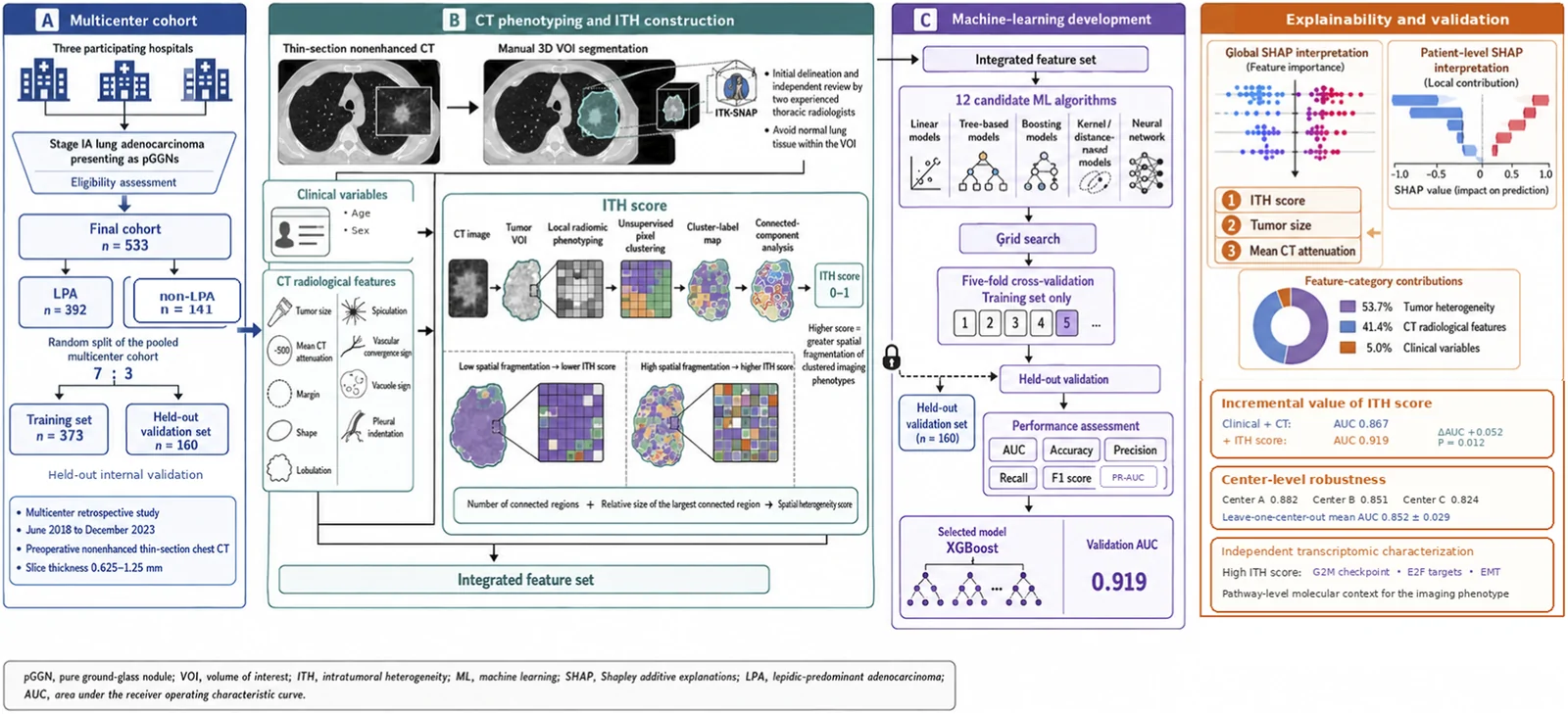
Study design and analytical workflow. The pooled multicenter cohort was divided into training and held-out internal validation cohorts. Model selection and hyperparameter optimization were confined to the training cohort using five-fold cross-validation, and the held-out cohort was evaluated only after model lock. The figure summarizes CT phenotyping and ITH construction, training-only machine-learning development, *post hoc* SHAP interpretation, incremental-value analysis, leave-one-center-out validation, and independent transcriptomic characterization. CT, computed tomography; ITH, intratumoral heterogeneity; LPA, lepidic-predominant adenocarcinoma; ML, machine learning; pGGN, pure ground-glass nodule; SHAP, Shapley additive explanations.

### CT acquisition and radiological assessment

CT scanners and acquisition parameters are summarized in Supplementary Table S1. Nonenhanced thin-section chest CT images were reviewed by two thoracic radiologists with more than 10 years of experience. Prespecified radiological variables were age, sex, tumor size (mm), mean CT attenuation (HU), margin, shape, lobulation, spiculation, vascular convergence sign, vacuole sign, and pleural indentation. Disagreements in qualitative CT assessment were resolved by consensus.

### Lesion segmentation and reproducibility

Tumors were manually segmented using ITK-SNAP (version 3.9.0). The contour was placed along the tumor boundary while avoiding adjacent normal tissue. For repeat-segmentation analysis, a representative subset of 100 lesions was segmented twice by one reader and independently by a second reader. Reproducibility was assessed for both the derived ITH score and the final XGBoost-predicted probability using intraclass correlation coefficients (ICCs) with 95% confidence intervals.

### ITH score calculation

The ITH score was calculated using the established ITHscore framework, which combines local radiomic phenotyping with global connected-region analysis (Li et al., 2023). For each pixel within the segmented tumor region, radiomic descriptors were extracted from a 2 × 2-pixel local neighborhood. A total of 104 local radiomics features were used, spanning first-order statistics, shape descriptors, GLCM, GLDM, GLRLM, GLSZM, and NGTDM classes; the complete feature list is provided in Supplementary Table S2. Local feature vectors were not z-standardized before clustering. K-means clustering with five clusters was applied to group pixels with similar local imaging phenotypes. Cluster labels were mapped back to tumor space, and spatially connected regions were identified using an 8-connectivity rule. The resulting connected-region pattern was summarized as the ITH score, with higher values indicating greater spatial fragmentation of clustered imaging phenotypes. Identical extraction parameters were used across centers. The implementation was based on the publicly available ITHscore Python package (https://pypi.org/project/ITHscore/).

### Machine-learning model development

Twelve supervised algorithms were evaluated: logistic regression, linear discriminant analysis, classification and regression tree, random forest, gradient boosting decision tree, adaptive boosting, extreme gradient boosting (XGBoost), LightGBM, CatBoost, support vector machine, k-nearest neighbor, and multilayer perceptron. The analysis used 12 prespecified predictors: age, sex, tumor size, mean CT attenuation, margin, shape, lobulation, spiculation, vascular convergence sign, vacuole sign, pleural indentation, and the ITH score.

All model selection and hyperparameter optimization were confined to the training cohort. Stratified five-fold cross-validation was used, and GridSearchCV optimized candidate hyperparameters with ROC-AUC as the scoring metric. Continuous variables were standardized using parameters fitted within the corresponding training folds for scale-sensitive algorithms; tree-based methods used unscaled input features. Binary CT signs were encoded as 0/1 and multicategory variables were one-hot encoded within the training pipeline. No class weighting was applied. Random state was used for data splitting, cross-validation, and model initialization. XGBoost achieved the highest training-set cross-validation AUC and was selected as the primary classifier before the held-out cohort was evaluated. The predictor set, preprocessing rules, hyperparameters, and probability threshold of 0.50 were then locked, and the final model was refitted on the entire training cohort. Complete parameter specifications are provided in Supplementary Table S3.

### Performance, incremental value, and center-level validation

The held-out validation cohort was evaluated once after model lock. Performance was summarized using ROC-AUC, accuracy, precision, sensitivity (recall), F1 score, specificity, negative predictive value (NPV), balanced accuracy, PR-AUC, and Brier score. Ninety-five percent confidence intervals were reported for the principal performance measures. The confusion matrix was also examined because non-LPA represented the clinically relevant positive class.

Incremental value of the ITH score was assessed using three prespecified models within the same XGBoost framework: M1, ITH score only; M2, clinical and conventional CT features; and M3, clinical and conventional CT features plus ITH score. M3 and M2 ROC-AUCs were compared using the paired DeLong test. To assess robustness to institution-level shifts, leave-one-center-out internal–external validation was performed by holding out each center in turn while using the remaining centers for model development.

### Model interpretation

SHAP values were not used for feature selection, hyperparameter optimization, or model selection. SHAP analysis was performed only after the final XGBoost model had been locked and was used exclusively for *post hoc* model interpretation. Global SHAP summaries quantified cohort-level model attribution, whereas local dependence plots characterized the direction of contributions for the leading predictors. SHAP values were interpreted as model attributions and not as causal biological effects.

### Independent transcriptomic characterization

To complement the stage I imaging analysis with molecular characterization, an independent radiogenomic analysis was performed using the publicly available NSCLC-Radiogenomics resource and its RNA-sequencing dataset GSE103584 (Zhou et al., 2018; Bakr et al., 2018). The resource contains paired thoracic imaging and molecular data from Stanford University and the VA Palo Alto Healthcare System. After matching available pathology and ITH information and restricting the analysis to pathological Tis/T1 adenocarcinoma, 43 cases were retained. This cohort was independent of the 533-patient machine-learning development and validation cohort. Cases were divided into high- and low-ITH-score groups using the cohort median ITH score; the cutoff was determined solely from the ITH distribution and without reference to transcriptomic outcomes.

Global expression structure was visualized using principal component analysis. Differential-expression analysis was performed using limma with high versus low ITH score as the coefficient of interest while adjusting for pathological T stage and patient affiliation (Ritchie et al., 2015). Genes were ranked by the moderated t statistic and analyzed by preranked gene set enrichment analysis (GSEA) using the MSigDB Hallmark collection (Subramanian et al., 2005; Liberzon et al., 2015). Positive normalized enrichment scores (NESs) indicate enrichment toward the High-ITH end of the ranked list. False-discovery rate (FDR) correction was applied for multiple testing.

### Statistical analysis

Statistical analyses were performed in Python 3.6.5 and R 4.4.1. Group comparisons used appropriate parametric or categorical tests. Statistical significance for conventional hypothesis testing was defined as two-sided P < 0.05. ROC analyses used pROC, and GSEA results were interpreted using FDR-adjusted significance. Brier scores were used as an overall measure of probabilistic prediction error.

## Results

### Patient characteristics

A total of 533 patients were included; 392 (73.5%) had LPA and 141 (26.5%) had non-LPA histology. The training cohort included 373 patients (LPA, 276; non-LPA, 97), and the held-out validation cohort included 160 patients (LPA, 116; non-LPA, 44). Baseline characteristics were similar between the training and validation cohorts (Table 1). Compared with LPA, non-LPA tumors more frequently showed irregular shape, vacuole sign, and pleural indentation and were associated with older age, larger tumor size, higher mean CT attenuation, and higher ITH score (Table 2).

**TABLE 1:** Clinical, tumor-heterogeneity, and radiological characteristics in the training and validation cohorts.

Variables	Training set (N = 373)	Validation set (N = 160)	Total (N = 533)	P value
Location				0.140
- RUL	125 (33.51%)	60 (37.50%)	185 (34.71%)	
- RLL	33 (8.85%)	8 (5.00%)	41 (7.69%)	
- RML	64 (17.16%)	17 (10.62%)	81 (15.20%)	
- LUL	103 (27.61%)	52 (32.50%)	155 (29.08%)	
- LLL	48 (12.87%)	23 (14.38%)	71 (13.32%)	
Margin	69 (18.50%)	22 (13.75%)	91 (17.07%)	0.226
Shape	110 (29.49%)	44 (27.50%)	154 (28.89%)	0.718
Lobulation	132 (35.39%)	57 (35.62%)	189 (35.46%)	1.000
Spiculation	101 (27.08%)	38 (23.75%)	139 (26.08%)	0.487
Vascular convergence sign	278 (74.53%)	125 (78.12%)	403 (75.61%)	0.438
Vacuole sign	47 (12.60%)	24 (15.00%)	71 (13.32%)	0.543
Pleural indentation	152 (40.75%)	54 (33.75%)	206 (38.65%)	0.154
Sex (female)	264 (70.78%)	112 (70.00%)	376 (70.54%)	0.939
Age (years)	56.00 [49.00; 66.00]	54.00 [46.00; 63.00]	56.00 [48.00; 65.00]	0.094
Tumor size (mm)	13.60 [10.20; 18.79]	12.21 [9.49; 17.60]	13.45 [9.85; 18.44]	0.136
Mean CT attenuation (HU)	−608.75 [-670.36; −522.91]	−614.00 [-678.81; −557.82]	−609.80 [-671.74; −534.64]	0.239
Pathologic diagnosis (non-LPA)	97 (26.01%)	44 (27.50%)	141 (26.45%)	0.801

Abbreviations: CT, computed tomography; ITH, intratumoral heterogeneity; LPA, lepidic-predominant adenocarcinoma.

**TABLE 2:** Clinical, ITH score, and radiological characteristics of LPA and non-LPA histology.

Variables	LPA (N = 392)	Non-LPA (N = 141)	Total (N = 533)	P value
Location				0.414
- RUL	140 (35.71%)	45 (31.91%)	185 (34.71%)	
- RLL	34 (8.67%)	7 (4.96%)	41 (7.69%)	
- RML	57 (14.54%)	24 (17.02%)	81 (15.20%)	
- LUL	108 (27.55%)	47 (33.33%)	155 (29.08%)	
- LLL	53 (13.52%)	18 (12.77%)	71 (13.32%)	
Margin	63 (16.07%)	28 (19.86%)	91 (17.07%)	0.371
Shape	93 (23.72%)	61 (43.26%)	154 (28.89%)	<0.001
Lobulation	134 (34.18%)	55 (39.01%)	189 (35.46%)	0.355
Spiculation	95 (24.23%)	44 (31.21%)	139 (26.08%)	0.132
Vascular convergence sign	289 (73.72%)	114 (80.85%)	403 (75.61%)	0.115
Vacuole sign	39 (9.95%)	32 (22.70%)	71 (13.32%)	<0.001
Pleural indentation	128 (32.65%)	78 (55.32%)	206 (38.65%)	<0.001
Sex (female)	286 (72.96%)	90 (63.83%)	376 (70.54%)	0.053
Age (years)	54.50 [46.00; 65.00]	57.00 [52.00; 66.00]	56.00 [48.00; 65.00]	0.005
Tumor size (mm)	11.53 [9.43; 15.81]	18.36 [15.23; 22.47]	13.45 [9.85; 18.44]	<0.001
Mean CT attenuation (HU)	−628.12 [-683.09; −560.43]	−549.86 [-624.13; −487.41]	−609.80 [-671.74; −534.64]	<0.001
ITH score	0.48 [0.34; 0.63]	0.72 [0.61; 0.80]	0.56 [0.39; 0.70]	<0.001

Abbreviations: CT, computed tomography; ITH, intratumoral heterogeneity; LPA, lepidic-predominant adenocarcinoma.

### Training-only model selection and held-out validation

Within the training cohort, XGBoost achieved the highest mean five-fold cross-validation AUC (0.908 ± 0.021), followed by gradient boosting decision tree (0.901 ± 0.024), AdaBoost (0.893 ± 0.026), random forest (0.885 ± 0.023), and LightGBM (0.882 ± 0.025) (Supplementary Table S4). XGBoost was therefore selected as the primary classifier before the held-out validation cohort was evaluated.

In the held-out validation cohort, XGBoost achieved an AUC of 0.919 (95% CI, 0.870–0.968), accuracy of 0.850 (95% CI, 0.789–0.901), precision of 0.857 (95% CI, 0.641–0.968), sensitivity of 0.545 (95% CI, 0.337–0.753), and F1 score of 0.667 (95% CI, 0.482–0.812) (Table 3; Figures 2, 3). The confusion matrix comprised 24 true positives, 20 false negatives, 4 false positives, and 112 true negatives, corresponding to specificity 0.966, NPV 0.848, balanced accuracy 0.756, and PR-AUC 0.824. The Brier score was 0.098. GBDT showed a numerically similar held-out AUC of 0.917 (95% CI, 0.868–0.966); classifier selection was based on training-set cross-validation rather than this small held-out difference.

**TABLE 3:** Held-out performance of the five highest-ranking machine-learning algorithms.

Model	AUC (95% CI)	Accuracy (95% CI)	Precision (95% CI)	Recall (95% CI)	F1 score (95% CI)	Brier score
Extreme gradient boosting	0.919 (0.870–0.968)	0.850 (0.789–0.901)	0.857 (0.641–0.968)	0.545 (0.337–0.753)	0.667 (0.482–0.812)	0.098
Gradient boosting decision tree	0.917 (0.868–0.966)	0.775 (0.704–0.836)	0.833 (0.516–0.979)	0.227 (0.078–0.454)	0.357 (0.142–0.548)	0.134
Adaptive boosting	0.907 (0.855–0.959)	0.813 (0.746–0.869)	0.719 (0.533–0.863)	0.523 (0.315–0.721)	0.605 (0.423–0.761)	0.112
Random forest	0.896 (0.840–0.952)	0.831 (0.767–0.883)	0.905 (0.696–0.988)	0.432 (0.234–0.647)	0.585 (0.376–0.755)	0.115
Light gradient boosting machine	0.895 (0.839–0.951)	0.813 (0.746–0.869)	0.769 (0.564–0.910)	0.455 (0.255–0.667)	0.571 (0.362–0.744)	0.118

Abbreviations: AUC, area under the receiver operating characteristic curve; CI, confidence interval. Brier score is an overall measure of probabilistic prediction error; lower values indicate lower error.

**FIGURE 2:**
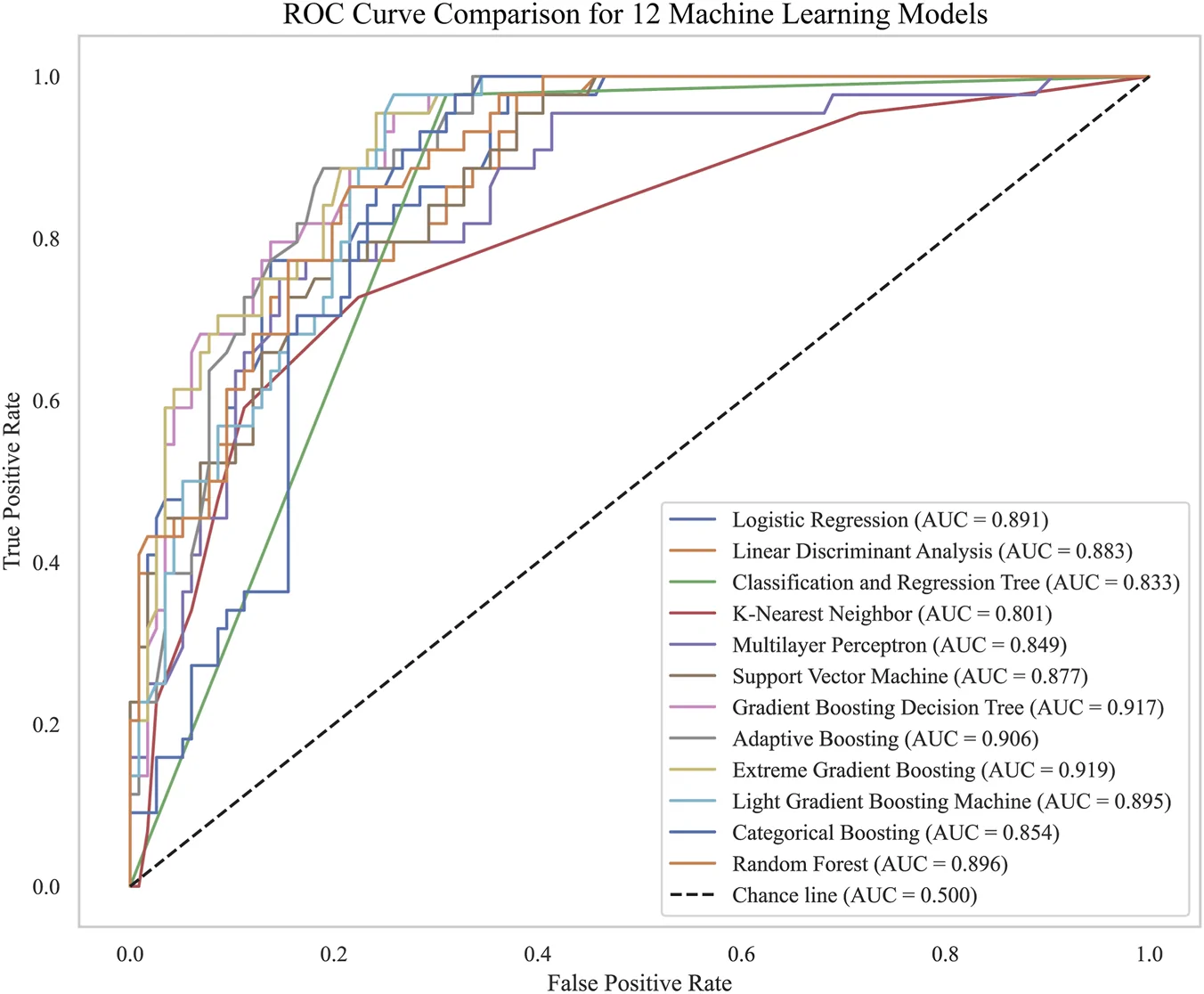
Receiver operating characteristic curves for the 12 machine-learning models in the held-out validation cohort. The curves are presented for descriptive benchmarking; classifier selection was completed within the training cohort before held-out evaluation. AUC, area under the receiver operating characteristic curve; ROC, receiver operating characteristic.

**FIGURE 3:**
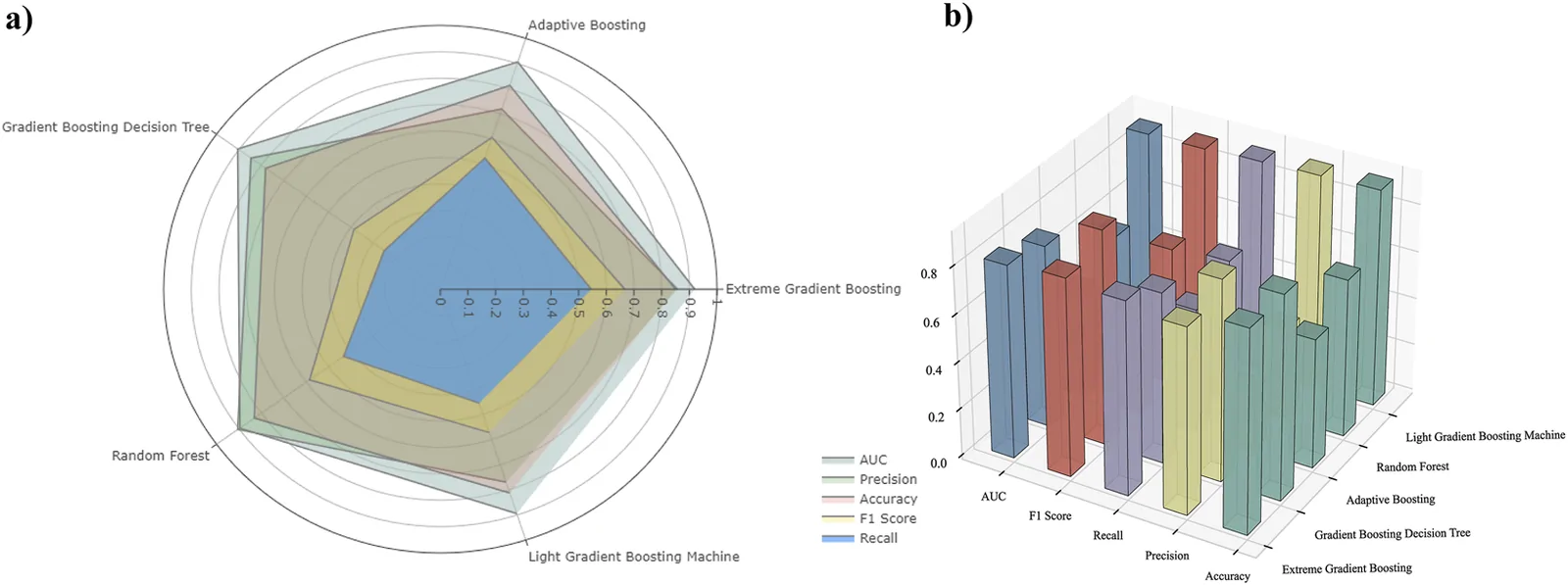
Held-out diagnostic performance of the five highest-ranking training-selected machine-learning algorithms. **(a)** Radar plot and **(b)** three-dimensional bar graph summarize AUC, accuracy, precision, recall, and F1 score. These held-out comparisons were descriptive and did not determine classifier selection. AUC, area under the receiver operating characteristic curve.

### Incremental value and center-level robustness

The ITH score-only model achieved an AUC of 0.834 (95% CI, 0.765–0.903). The clinical/CT model achieved an AUC of 0.867 (95% CI, 0.806–0.928), whereas addition of the ITH score increased the AUC to 0.919 (95% CI, 0.870–0.968), an absolute increase of 0.052 (paired DeLong P = 0.012). PR-AUC increased from 0.731 to 0.824 and Brier score decreased from 0.126 to 0.098 (Supplementary Table S5).

In leave-one-center-out internal–external validation, AUCs were 0.882 for Center A, 0.851 for Center B, and 0.824 for Center C, with a mean of 0.852 ± 0.029. Corresponding specificities remained high (0.907–0.942), whereas sensitivities ranged from 0.417 to 0.491, indicating that discrimination generalized better than the fixed operating threshold (Supplementary Table S6).

### Segmentation reproducibility

In 100 repeat-segmented lesions, the ITH score showed an intra-observer ICC of 0.91 (95% CI, 0.87–0.94) and an inter-observer ICC of 0.86 (95% CI, 0.80–0.90). XGBoost-predicted probabilities were similarly stable, with intra-observer and inter-observer ICCs of 0.94 (95% CI, 0.91–0.96) and 0.90 (95% CI, 0.85–0.93), respectively (Supplementary Table S7).

### Post-hoc model interpretation

Post-hoc SHAP analysis of the locked XGBoost model showed that the ITH score was the dominant predictor, followed by tumor size and mean CT attenuation (Figure 4). Tumor heterogeneity accounted for 53.7% of total model attribution, CT radiological features for 41.4%, and clinical variables for 5.0%. Local SHAP dependence plots showed descriptive changes in attribution around ITH scores >0.55, tumor sizes >13 mm, and mean CT attenuation > −610 HU (Figure 5). These were not prespecified clinical thresholds and should not be interpreted as causal cutoffs.

**FIGURE 4:**
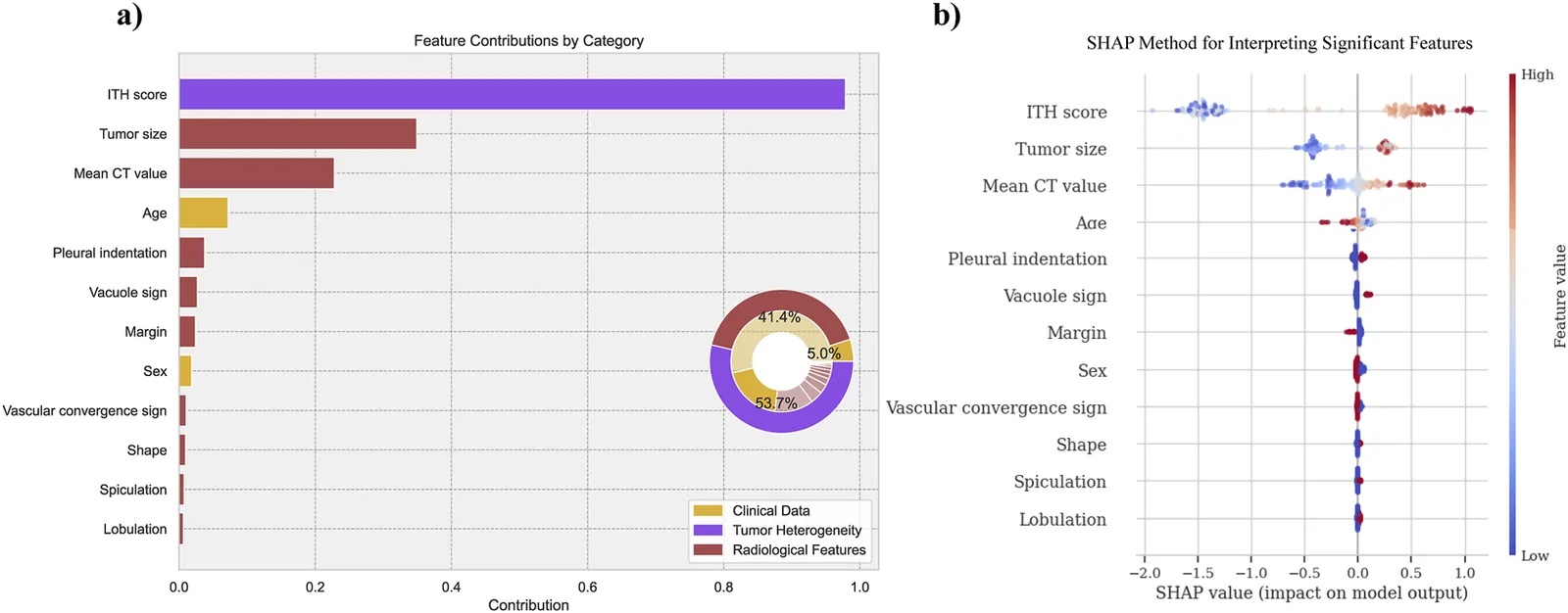
Post-hoc global SHAP interpretation of the locked XGBoost model. **(a)** Relative feature contributions by category and individual predictor. **(b)** SHAP beeswarm plot showing the direction and magnitude of model attribution. SHAP analysis was used only after model lock and was not used for feature selection or hyperparameter optimization. CT, computed tomography; ITH, intratumoral heterogeneity; SHAP, Shapley additive explanations.

**FIGURE 5:**
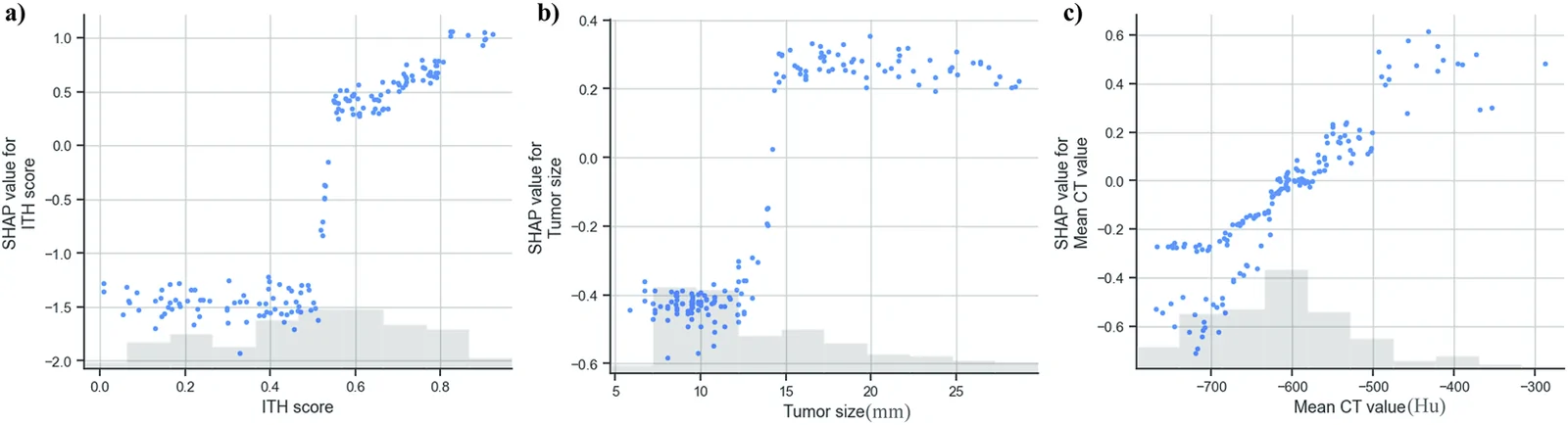
Local SHAP dependence plots for the leading predictors: **(a)** ITH score, **(b)** tumor size, and **(c)** mean CT attenuation. Positive SHAP values shift model output toward the non-LPA class. The observed patterns are descriptive and do not represent prespecified clinical thresholds. CT, computed tomography; HU, Hounsfield unit; ITH, intratumoral heterogeneity; LPA, lepidic-predominant adenocarcinoma; SHAP, Shapley additive explanations.

### Transcriptomic characterization of the ITH phenotype

The independent radiogenomic analysis included 43 pathological Tis/T1 adenocarcinomas, divided at the cohort median into high- and low-ITH-score groups. PCA showed partial overlap rather than complete separation of the two groups, indicating that ITH did not define a globally distinct transcriptomic subtype (Figures 6A,B). The top-ranked differential-expression heatmap nevertheless showed coordinated expression patterns across the high- and low-ITH-score groups (Figure 6C).

**FIGURE 6:**
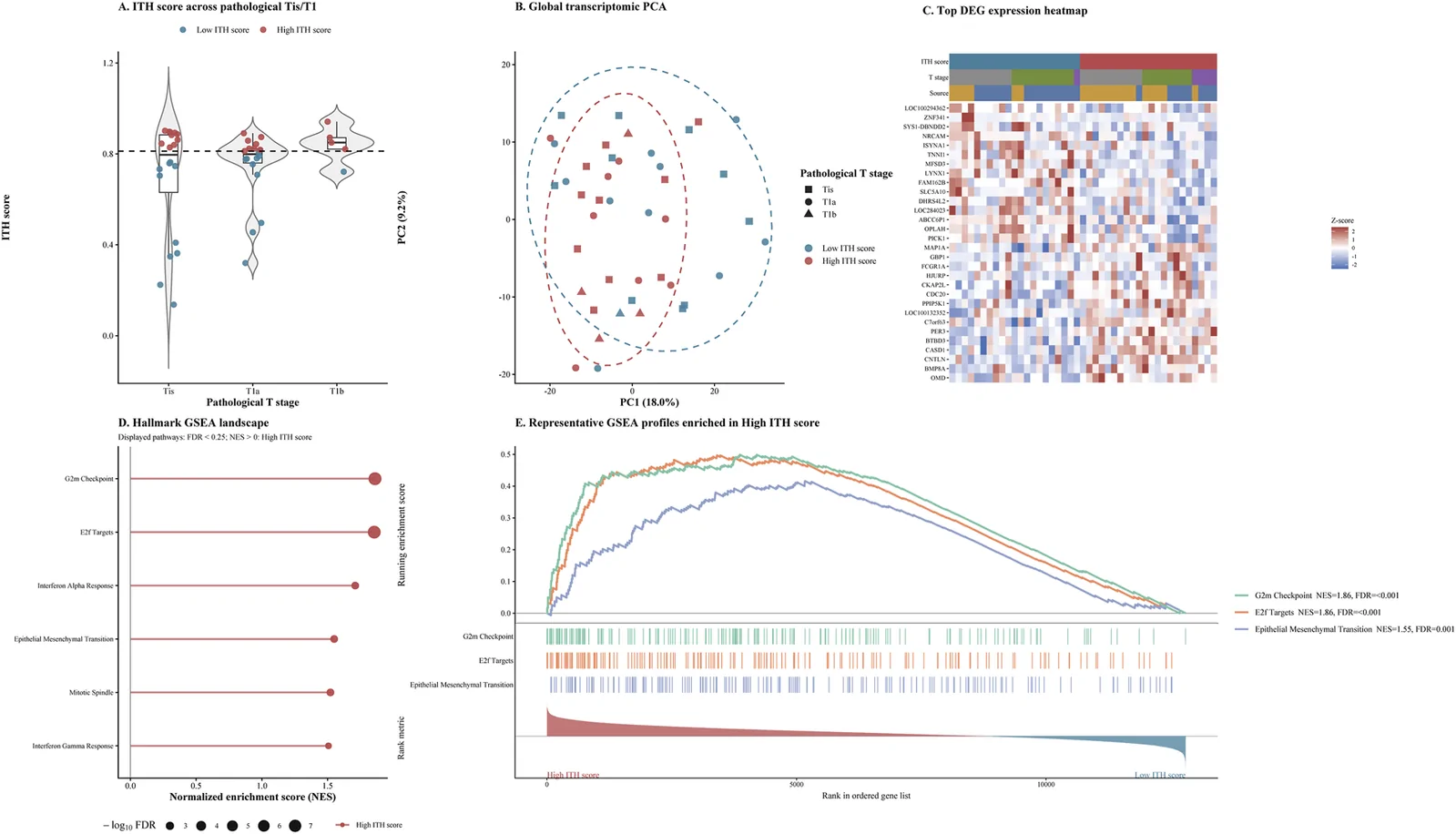
Transcriptomic programs associated with the ITH score in the independent pathological Tis/T1 lung adenocarcinoma radiogenomic cohort. **(A)** Distribution of ITH score across pathological Tis/T1 categories and median-based high/low ITH-score grouping. **(B)** PCA of global transcriptomic profiles. **(C)** Heatmap of top-ranked differential-expression patterns. **(D)** Hallmark GSEA landscape; positive NES denotes enrichment toward a high ITH score. **(E)** Representative GSEA profiles for G2M checkpoint, E2F targets, and epithelial–mesenchymal transition. FDR, false-discovery rate; GSEA, gene set enrichment analysis; ITH, intratumoral heterogeneity; NES, normalized enrichment score; PCA, principal component analysis.

At the pathway level, Hallmark GSEA identified coordinated positive enrichment toward a high ITH score, including G2M checkpoint, E2F targets, mitotic spindle, epithelial–mesenchymal transition, interferon alpha response, and interferon gamma response (Figure 6D). Representative enrichment profiles showed G2M checkpoint (NES = 1.86, FDR <0.001), E2F targets (NES = 1.86, FDR <0.001), and epithelial–mesenchymal transition (NES = 1.55, FDR = 0.001) as prominent high-ITH-score programs (Figure 6E; Supplementary Table S8). These findings support an association between the imaging-derived ITH phenotype and coordinated proliferative, cell-cycle, phenotypic-plasticity, and interferon-related transcriptional programs.

## Discussion

This multicenter study provides convergent evidence that CT-derived intratumoral heterogeneity adds information to pGGN histological subtype prediction beyond routine clinical and CT features. Training-set cross-validation identified XGBoost before held-out evaluation, and the locked model achieved an AUC of 0.919 in the validation cohort. More importantly, the contribution of the ITH score was demonstrated directly rather than inferred solely from feature attribution: adding ITH to clinical and CT features increased AUC by 0.052, increased PR-AUC by 0.093, and reduced the Brier score by 0.028.

The operating-point results also require a balanced interpretation. The model showed high specificity (0.966) and PPV (0.857) for non-LPA disease, but sensitivity was only 0.545 at the prespecified 0.50 threshold, meaning that a substantial proportion of non-LPA lesions were missed. Thus, the model should not be considered a stand-alone rule-out test or a substitute for pathological diagnosis. The leave-one-center-out analysis reinforces this point: discrimination remained reasonably stable across centers, whereas sensitivity decreased at the fixed threshold. Prospective work should therefore focus not only on AUC but also on threshold transportability and the clinical consequences of false-negative predictions.

The repeat-segmentation analysis addresses another important concern for a spatial heterogeneity metric. Both the ITH score and final predicted probability showed good-to-excellent intra- and inter-observer ICCs. These findings suggest that the principal signal was not dominated by small contour perturbations, although fully automated segmentation and prospective reproducibility studies remain desirable.

The broader relevance of spatially explicit heterogeneity quantification is supported by related work across several tumor types. Imaging-derived heterogeneity approaches have been explored in locally advanced nasopharyngeal carcinoma, colorectal cancer, breast cancer, and hepatocellular carcinoma, with reported associations involving prognosis, invasive phenotypes, treatment-related outcomes, or tumor microenvironmental features (Li et al., 2026; Zhou et al., 2026; Zuo et al., 2026d; Zuo et al., 2026b; Zhao J. et al., 2026). These cross-tumor applications support spatial heterogeneity as a general imaging concept while emphasizing that the biological meaning of an ITH score remains disease- and endpoint-specific.

The independent transcriptomic analysis provides a separate layer of biological characterization. A high ITH score was associated with coordinated enrichment of G2M checkpoint, E2F targets, mitotic spindle, EMT, and interferon-related programs. The convergence of G2M, E2F, and mitotic-spindle signals is consistent with a more proliferative and cell-cycle-active tumor state, in keeping with the central role of sustained proliferative signaling in malignant progression (Hanahan, 2022). EMT enrichment further suggests increased phenotypic plasticity and architectural remodeling, processes that permit transitions across epithelial–mesenchymal states and are closely linked to tumor progression, invasion, metastasis, and treatment resistance (Haerinck et al., 2023; Gu et al., 2023). The enrichment of interferon-alpha and interferon-gamma response programs may additionally reflect differences in the tumor–immune context, as both type I and type II interferon signaling exert context-dependent effects on antitumor immunity, immune escape, and treatment response within the tumor microenvironment (Gocher et al., 2022; Holicek et al., 2024). These findings should be interpreted as associations rather than evidence that the ITH score directly measures molecular heterogeneity or that imaging heterogeneity causally activates these pathways.

SHAP was restricted to *post hoc* interpretation and was not used for feature selection or model optimization. This separation allows feature attribution to improve transparency without introducing outcome-informed selection outside the training process. The observed SHAP dependence patterns remain descriptive and should not be treated as universal clinical thresholds.

### Limitations

Several limitations remain. First, this was a retrospective study. Although leave-one-center-out internal–external validation was added, a fully independent prospective external cohort was not available. Scanner and reconstruction differences may still affect local radiomic measurements.

Second, the non-LPA group was heterogeneous, and the retrospective pathology reports did not permit reliable subdivision of every non-LPA tumor into acinar-, papillary-, micropapillary-, and solid-predominant categories. The binary comparison therefore does not resolve differences among individual non-LPA patterns.

Third, class imbalance and the moderate sensitivity for non-LPA limit interpretation at the prespecified 0.50 threshold. The Brier score quantified overall probabilistic prediction error, but formal calibration and decision-curve analyses were beyond the scope of this retrospective study. Prospective work should therefore evaluate probability calibration, threshold transportability, and net benefit before implementation in clinical workflows.

Finally, the transcriptomic analysis was exploratory and based on an independent public radiogenomic cohort with a modest sample size. It supports biological plausibility at the pathway level but does not establish causality or validate the ITH score as a direct molecular surrogate.

## Conclusion

An explainable XGBoost model integrating CT-derived ITH with routine clinical and CT features showed favorable discrimination for differentiating LPA from non-LPA histology in pGGNs. The ITH score provided statistically significant incremental predictive information beyond clinical and CT features, demonstrated good repeat-segmentation reproducibility, and retained useful discrimination in center-level validation. Independent transcriptomic analysis further associated high ITH with cell-cycle/proliferative, EMT, and interferon-related programs. The moderate sensitivity and retrospective design support continued prospective external validation rather than immediate clinical use.

## Data Availability

The raw data supporting the conclusions of this article will be made available by the authors, without undue reservation.
