# Supplementary material for: Explainable machine learning integrating CT-derived intratumoral heterogeneity for histological subtype prediction in pure ground-glass lung adenocarcinoma: multicenter validation and transcriptomic characterization
Source: Front Mol Biosci. 2026 Oct 12;13:1958127. doi: 10.3389/fmolb.2026.1958127 (PMC13696266; doi:10.3389/fmolb.2026.1958127)
Supplement: Supplementary file 1 [file Table1.docx]

**Supplementary Tables**

## Supplementary Table S1. CT scanners and acquisition parameters across participating centers

| **Item** | **Description** |
| --- | --- |
| Scanner and acquisition setting | Preoperative chest CT scans were conducted using multidetector CT technology. Center A used the uCT550 or CT760 scanners (United Imaging Healthcare, Shanghai, China), Center B used the Siemens Somatom Force CT scanner (Siemens Healthineers, Erlangen, Germany), and Center C used the Philips Brilliance iCT scanner (Philips Healthcare, Best, the Netherlands). |
| Imaging protocol | Helical scanning was performed with patients supine during breath-holding, from the lung apex to below the costophrenic angle. Parameters included tube voltage 120 kV, tube current-time product 180–280 mA, matrix 512 × 512, and reconstructed section thickness 0.625–1.25 mm. |

Note: Participating sites are designated Center A, Center B, and Center C in the acquisition summary.

## Supplementary Table S2. Local radiomic features used for calculation of the ITH score

| **No.** | **Category** | **PyRadiomics class prefix** | **Feature name** | **Full local feature key** |
| --- | --- | --- | --- | --- |
| 1 | First-order statistics | original_firstorder | 10Percentile | original_firstorder_10Percentile |
| 2 | First-order statistics | original_firstorder | 90Percentile | original_firstorder_90Percentile |
| 3 | First-order statistics | original_firstorder | Energy | original_firstorder_Energy |
| 4 | First-order statistics | original_firstorder | Entropy | original_firstorder_Entropy |
| 5 | First-order statistics | original_firstorder | InterquartileRange | original_firstorder_InterquartileRange |
| 6 | First-order statistics | original_firstorder | Kurtosis | original_firstorder_Kurtosis |
| 7 | First-order statistics | original_firstorder | Maximum | original_firstorder_Maximum |
| 8 | First-order statistics | original_firstorder | Mean | original_firstorder_Mean |
| 9 | First-order statistics | original_firstorder | MeanAbsoluteDeviation | original_firstorder_MeanAbsoluteDeviation |
| 10 | First-order statistics | original_firstorder | Median | original_firstorder_Median |
| 11 | First-order statistics | original_firstorder | Minimum | original_firstorder_Minimum |
| 12 | First-order statistics | original_firstorder | Range | original_firstorder_Range |
| 13 | First-order statistics | original_firstorder | RobustMeanAbsoluteDeviation | original_firstorder_RobustMeanAbsoluteDeviation |
| 14 | First-order statistics | original_firstorder | RootMeanSquared | original_firstorder_RootMeanSquared |
| 15 | First-order statistics | original_firstorder | Skewness | original_firstorder_Skewness |
| 16 | First-order statistics | original_firstorder | StandardDeviation | original_firstorder_StandardDeviation |
| 17 | First-order statistics | original_firstorder | TotalEnergy | original_firstorder_TotalEnergy |
| 18 | First-order statistics | original_firstorder | Uniformity | original_firstorder_Uniformity |
| 19 | First-order statistics | original_firstorder | Variance | original_firstorder_Variance |
| 20 | Shape / shape2D | original_shape2D | Elongation | original_shape2D_Elongation |
| 21 | Shape / shape2D | original_shape2D | MajorAxisLength | original_shape2D_MajorAxisLength |
| 22 | Shape / shape2D | original_shape2D | MaximumDiameter | original_shape2D_MaximumDiameter |
| 23 | Shape / shape2D | original_shape2D | MeshSurface | original_shape2D_MeshSurface |
| 24 | Shape / shape2D | original_shape2D | MinorAxisLength | original_shape2D_MinorAxisLength |
| 25 | Shape / shape2D | original_shape2D | Perimeter | original_shape2D_Perimeter |
| 26 | Shape / shape2D | original_shape2D | PerimeterSurfaceRatio | original_shape2D_PerimeterSurfaceRatio |
| 27 | Shape / shape2D | original_shape2D | PixelSurface | original_shape2D_PixelSurface |
| 28 | Shape / shape2D | original_shape2D | Sphericity | original_shape2D_Sphericity |
| 29 | Shape / shape2D | original_shape2D | SurfaceVolumeRatio | original_shape2D_SurfaceVolumeRatio |
| 30 | GLCM | original_glcm | Autocorrelation | original_glcm_Autocorrelation |
| 31 | GLCM | original_glcm | ClusterProminence | original_glcm_ClusterProminence |
| 32 | GLCM | original_glcm | ClusterShade | original_glcm_ClusterShade |
| 33 | GLCM | original_glcm | ClusterTendency | original_glcm_ClusterTendency |
| 34 | GLCM | original_glcm | Contrast | original_glcm_Contrast |
| 35 | GLCM | original_glcm | Correlation | original_glcm_Correlation |
| 36 | GLCM | original_glcm | DifferenceAverage | original_glcm_DifferenceAverage |
| 37 | GLCM | original_glcm | DifferenceEntropy | original_glcm_DifferenceEntropy |
| 38 | GLCM | original_glcm | DifferenceVariance | original_glcm_DifferenceVariance |
| 39 | GLCM | original_glcm | Id | original_glcm_Id |
| 40 | GLCM | original_glcm | Idm | original_glcm_Idm |
| 41 | GLCM | original_glcm | Idmn | original_glcm_Idmn |
| 42 | GLCM | original_glcm | Idn | original_glcm_Idn |
| 43 | GLCM | original_glcm | Imc1 | original_glcm_Imc1 |
| 44 | GLCM | original_glcm | Imc2 | original_glcm_Imc2 |
| 45 | GLCM | original_glcm | InverseVariance | original_glcm_InverseVariance |
| 46 | GLCM | original_glcm | JointAverage | original_glcm_JointAverage |
| 47 | GLCM | original_glcm | JointEnergy | original_glcm_JointEnergy |
| 48 | GLCM | original_glcm | JointEntropy | original_glcm_JointEntropy |
| 49 | GLCM | original_glcm | MCC | original_glcm_MCC |
| 50 | GLCM | original_glcm | MaximumProbability | original_glcm_MaximumProbability |
| 51 | GLCM | original_glcm | SumAverage | original_glcm_SumAverage |
| 52 | GLCM | original_glcm | SumEntropy | original_glcm_SumEntropy |
| 53 | GLCM | original_glcm | SumSquares | original_glcm_SumSquares |
| 54 | GLDM | original_gldm | DependenceEntropy | original_gldm_DependenceEntropy |
| 55 | GLDM | original_gldm | DependenceNonUniformity | original_gldm_DependenceNonUniformity |
| 56 | GLDM | original_gldm | DependenceNonUniformityNormalized | original_gldm_DependenceNonUniformityNormalized |
| 57 | GLDM | original_gldm | DependenceVariance | original_gldm_DependenceVariance |
| 58 | GLDM | original_gldm | GrayLevelNonUniformity | original_gldm_GrayLevelNonUniformity |
| 59 | GLDM | original_gldm | GrayLevelVariance | original_gldm_GrayLevelVariance |
| 60 | GLDM | original_gldm | HighGrayLevelEmphasis | original_gldm_HighGrayLevelEmphasis |
| 61 | GLDM | original_gldm | LargeDependenceEmphasis | original_gldm_LargeDependenceEmphasis |
| 62 | GLDM | original_gldm | LargeDependenceHighGrayLevelEmphasis | original_gldm_LargeDependenceHighGrayLevelEmphasis |
| 63 | GLDM | original_gldm | LargeDependenceLowGrayLevelEmphasis | original_gldm_LargeDependenceLowGrayLevelEmphasis |
| 64 | GLDM | original_gldm | LowGrayLevelEmphasis | original_gldm_LowGrayLevelEmphasis |
| 65 | GLDM | original_gldm | SmallDependenceEmphasis | original_gldm_SmallDependenceEmphasis |
| 66 | GLDM | original_gldm | SmallDependenceHighGrayLevelEmphasis | original_gldm_SmallDependenceHighGrayLevelEmphasis |
| 67 | GLDM | original_gldm | SmallDependenceLowGrayLevelEmphasis | original_gldm_SmallDependenceLowGrayLevelEmphasis |
| 68 | GLRLM | original_glrlm | GrayLevelNonUniformity | original_glrlm_GrayLevelNonUniformity |
| 69 | GLRLM | original_glrlm | GrayLevelNonUniformityNormalized | original_glrlm_GrayLevelNonUniformityNormalized |
| 70 | GLRLM | original_glrlm | GrayLevelVariance | original_glrlm_GrayLevelVariance |
| 71 | GLRLM | original_glrlm | HighGrayLevelRunEmphasis | original_glrlm_HighGrayLevelRunEmphasis |
| 72 | GLRLM | original_glrlm | LongRunEmphasis | original_glrlm_LongRunEmphasis |
| 73 | GLRLM | original_glrlm | LongRunHighGrayLevelEmphasis | original_glrlm_LongRunHighGrayLevelEmphasis |
| 74 | GLRLM | original_glrlm | LongRunLowGrayLevelEmphasis | original_glrlm_LongRunLowGrayLevelEmphasis |
| 75 | GLRLM | original_glrlm | LowGrayLevelRunEmphasis | original_glrlm_LowGrayLevelRunEmphasis |
| 76 | GLRLM | original_glrlm | RunEntropy | original_glrlm_RunEntropy |
| 77 | GLRLM | original_glrlm | RunLengthNonUniformity | original_glrlm_RunLengthNonUniformity |
| 78 | GLRLM | original_glrlm | RunLengthNonUniformityNormalized | original_glrlm_RunLengthNonUniformityNormalized |
| 79 | GLRLM | original_glrlm | RunPercentage | original_glrlm_RunPercentage |
| 80 | GLRLM | original_glrlm | RunVariance | original_glrlm_RunVariance |
| 81 | GLRLM | original_glrlm | ShortRunEmphasis | original_glrlm_ShortRunEmphasis |
| 82 | GLRLM | original_glrlm | ShortRunHighGrayLevelEmphasis | original_glrlm_ShortRunHighGrayLevelEmphasis |
| 83 | GLRLM | original_glrlm | ShortRunLowGrayLevelEmphasis | original_glrlm_ShortRunLowGrayLevelEmphasis |
| 84 | GLSZM | original_glszm | GrayLevelNonUniformity | original_glszm_GrayLevelNonUniformity |
| 85 | GLSZM | original_glszm | GrayLevelNonUniformityNormalized | original_glszm_GrayLevelNonUniformityNormalized |
| 86 | GLSZM | original_glszm | GrayLevelVariance | original_glszm_GrayLevelVariance |
| 87 | GLSZM | original_glszm | HighGrayLevelZoneEmphasis | original_glszm_HighGrayLevelZoneEmphasis |
| 88 | GLSZM | original_glszm | LargeAreaEmphasis | original_glszm_LargeAreaEmphasis |
| 89 | GLSZM | original_glszm | LargeAreaHighGrayLevelEmphasis | original_glszm_LargeAreaHighGrayLevelEmphasis |
| 90 | GLSZM | original_glszm | LargeAreaLowGrayLevelEmphasis | original_glszm_LargeAreaLowGrayLevelEmphasis |
| 91 | GLSZM | original_glszm | LowGrayLevelZoneEmphasis | original_glszm_LowGrayLevelZoneEmphasis |
| 92 | GLSZM | original_glszm | SizeZoneNonUniformity | original_glszm_SizeZoneNonUniformity |
| 93 | GLSZM | original_glszm | SizeZoneNonUniformityNormalized | original_glszm_SizeZoneNonUniformityNormalized |
| 94 | GLSZM | original_glszm | SmallAreaEmphasis | original_glszm_SmallAreaEmphasis |
| 95 | GLSZM | original_glszm | SmallAreaHighGrayLevelEmphasis | original_glszm_SmallAreaHighGrayLevelEmphasis |
| 96 | GLSZM | original_glszm | SmallAreaLowGrayLevelEmphasis | original_glszm_SmallAreaLowGrayLevelEmphasis |
| 97 | GLSZM | original_glszm | ZoneEntropy | original_glszm_ZoneEntropy |
| 98 | GLSZM | original_glszm | ZonePercentage | original_glszm_ZonePercentage |
| 99 | GLSZM | original_glszm | ZoneVariance | original_glszm_ZoneVariance |
| 100 | NGTDM | original_ngtdm | Busyness | original_ngtdm_Busyness |
| 101 | NGTDM | original_ngtdm | Coarseness | original_ngtdm_Coarseness |
| 102 | NGTDM | original_ngtdm | Complexity | original_ngtdm_Complexity |
| 103 | NGTDM | original_ngtdm | Contrast | original_ngtdm_Contrast |
| 104 | NGTDM | original_ngtdm | Strength | original_ngtdm_Strength |

Abbreviations: GLCM, gray-level co-occurrence matrix; GLDM, gray-level dependence matrix; GLRLM, gray-level run-length matrix; GLSZM, gray-level size-zone matrix; NGTDM, neighboring gray-tone difference matrix; ITH, intratumoral heterogeneity.

## Supplementary Table S3. Reproducibility specifications of the machine-learning pipeline

| **Component** | **Specification** |
| --- | --- |
| Study design | Retrospective multicenter; 533 patients; stratified 7:3 training/held-out validation split by histological subtype. |
| Random seed | 42 (applied to data splitting, cross-validation, and model initialization). |
| Outcome | Binary: LPA (0) vs non-LPA histology (1). |
| Training set | n = 373 (LPA = 276, non-LPA = 97; positive rate 26.0%). |
| Validation set | n = 160 (LPA = 116, non-LPA = 44; positive rate 27.5%). |
| Cross-validation | Five-fold stratified CV within the training cohort; held-out validation was unused for model selection. |
| Missing data | No missing values in the final analytic cohort; cases with incomplete CT feature assessment were excluded during data-quality review before model development. |
| Categorical encoding | Binary CT signs encoded as 0/1; multicategory variables (margin and shape) one-hot encoded within the training pipeline. |
| Scaling | StandardScaler (mean = 0, SD = 1) fitted within training folds for age, tumor size, mean CT attenuation, and ITH score in scale-sensitive algorithms; tree-based models used raw features. |
| Class weighting | No class weighting; scale_pos_weight = 1 for XGBoost and class_weight = None for scikit-learn models. Primary decision threshold = 0.50. |
| Feature list (12 variables) | Clinical (2): age, sex; CT quantitative (2): tumor size, mean CT attenuation; CT qualitative (7): margin, shape, lobulation, spiculation, vascular convergence sign, vacuole sign, pleural indentation; ITH (1): ITH score. |
| Hyperparameter search | GridSearchCV with five-fold stratified CV; scoring metric = ROC-AUC; final estimator refitted on the complete training cohort using selected parameters. |
| XGBoost hyperparameter grid | n_estimators [100, 200, 300, 500]; max_depth [3, 5, 7, 9]; learning_rate [0.01, 0.05, 0.1, 0.2]; subsample [0.7, 0.8, 0.9, 1.0]; colsample_bytree [0.7, 0.8, 0.9, 1.0]; min_child_weight [1, 3, 5]; reg_alpha [0, 0.01, 0.1, 1.0]; reg_lambda [0.1, 1.0, 10.0]. |
| Selected XGBoost hyperparameters | n_estimators = 300; max_depth = 5; learning_rate = 0.05; subsample = 0.8; colsample_bytree = 0.8; min_child_weight = 3; reg_alpha = 0.1; reg_lambda = 1.0; scale_pos_weight = 1; objective = binary:logistic; eval_metric = logloss. |
| Software — Python | Python 3.6.5; scikit-learn 0.24.2; xgboost 1.5.0; numpy 1.19.5; pandas 1.1.5; shap 0.40.0. |
| Software — R | R 4.4.1; pROC 1.18.0; compareGroups 4.7.0. |
| SHAP role | Post-hoc interpretation only after model lock; not used for feature selection, model selection, or hyperparameter optimization. |
| Reproducibility | All random processes seeded; code and parameter logs archived; held-out validation evaluated once after final model lock. |

## Supplementary Table S4. Training-set cross-validation performance of the 12 candidate algorithms

| **Algorithm** | **AUC (mean ± SD)** | **Accuracy (mean ± SD)** |
| --- | --- | --- |
| Extreme Gradient Boosting | 0.908 ± 0.021 | 0.842 ± 0.028 |
| Gradient Boosting Decision Tree | 0.901 ± 0.024 | 0.815 ± 0.031 |
| Adaptive Boosting | 0.893 ± 0.026 | 0.827 ± 0.029 |
| Random Forest | 0.885 ± 0.023 | 0.831 ± 0.026 |
| Light Gradient Boosting Machine | 0.882 ± 0.025 | 0.819 ± 0.030 |
| Categorical Boosting | 0.871 ± 0.028 | 0.810 ± 0.033 |
| Support Vector Machine | 0.846 ± 0.032 | 0.794 ± 0.035 |
| Multilayer Perceptron | 0.838 ± 0.035 | 0.786 ± 0.038 |
| Logistic Regression | 0.821 ± 0.030 | 0.775 ± 0.032 |
| Linear Discriminant Analysis | 0.812 ± 0.029 | 0.768 ± 0.034 |
| Classification and Regression Tree | 0.774 ± 0.041 | 0.742 ± 0.042 |
| K-Nearest Neighbor | 0.758 ± 0.044 | 0.728 ± 0.045 |

Five-fold stratified cross-validation was performed exclusively within the training cohort (n = 373). Hyperparameter optimization used GridSearchCV with ROC-AUC as the scoring criterion. XGBoost was selected as the primary classifier according to training-set cross-validation performance before the held-out cohort was evaluated. Performance of other high-ranking algorithms in the held-out cohort was reported only for descriptive benchmarking and did not contribute to model selection.

## Supplementary Table S5. Incremental predictive value of the ITH score

| **Metric** | **M1: ITH score only** | **M2: Clinical + CT** | **M3: Clinical + CT + ITH score** | **Δ (M3 − M2)** | **P value (DeLong)** |
| --- | --- | --- | --- | --- | --- |
| AUC (95% CI) | 0.834 (0.765–0.903) | 0.867 (0.806–0.928) | 0.919 (0.870–0.968) | +0.052 | 0.012 |
| PR-AUC | 0.652 | 0.731 | 0.824 | +0.093 | — |
| Brier score | 0.148 | 0.126 | 0.098 | −0.028 | — |

Abbreviations: AUC, area under the receiver operating characteristic curve; CI, confidence interval; ITH, intratumoral heterogeneity; PR-AUC, area under the precision–recall curve. Positive Δ for AUC/PR-AUC and negative Δ for Brier score indicate improvement. The paired DeLong test was prespecified for the M3 versus M2 AUC comparison.

## Supplementary Table S6. Leave-one-center-out internal–external validation

| **Held-out center** | **n** | **Non-LPA (n)** | **AUC** | **PR-AUC** | **Sensitivity** | **Specificity** | **Brier score** |
| --- | --- | --- | --- | --- | --- | --- | --- |
| Center A | 210 | 55 | 0.882 | 0.756 | 0.491 | 0.942 | 0.112 |
| Center B | 180 | 50 | 0.851 | 0.703 | 0.440 | 0.923 | 0.128 |
| Center C | 143 | 36 | 0.824 | 0.658 | 0.417 | 0.907 | 0.141 |
| Mean ± SD | — | — | 0.852 ± 0.029 | 0.706 ± 0.049 | 0.449 ± 0.037 | 0.924 ± 0.018 | 0.127 ± 0.015 |

## Supplementary Table S7. Segmentation reproducibility (n = 100)

| **Metric** | **Intra-observer (R1 vs R1′)** | **Inter-observer (R1 vs R2)** |
| --- | --- | --- |
| ITH score ICC (95% CI) | 0.91 (0.87–0.94) | 0.86 (0.80–0.90) |
| XGBoost predicted probability ICC (95% CI) | 0.94 (0.91–0.96) | 0.90 (0.85–0.93) |

Abbreviations: CI, confidence interval; ICC, intraclass correlation coefficient; ITH, intratumoral heterogeneity; XGBoost, extreme gradient boosting.

## Supplementary Table S8. Representative Hallmark GSEA results associated with high ITH score

| **Hallmark pathway** | **NES** | **FDR** | **Interpretive theme** |
| --- | --- | --- | --- |
| G2M Checkpoint | 1.86 | <0.001 | Cell-cycle/proliferative activity |
| E2F Targets | 1.86 | <0.001 | Cell-cycle and DNA-replication program |
| Epithelial Mesenchymal Transition | 1.55 | 0.001 | Phenotypic plasticity / EMT-related program |
